# Genomic evaluation of milk yield in a smallholder crossbred dairy production system in India

**DOI:** 10.1186/s12711-021-00667-6

**Published:** 2021-09-10

**Authors:** Mohammad Al Kalaldeh, Marimuthu Swaminathan, Yuvraj Gaundare, Sachin Joshi, Hassan Aliloo, Eva M. Strucken, Vincent Ducrocq, John P. Gibson

**Affiliations:** 1 Centre for Genetic Analysis and Applications, School of Environmental and Rural Science, University of New England, Armidale, NSW 2350 Australia; 2 BAIF Development Research Foundation and Central Research Station, Uruli Kanchan, Pune, Maharashtra 412202 India; 3 Université Paris-Saclay, INRAE, AgroParisTech, UMR GABI, 78350 Jouy-en-Josas, France

## Abstract

**Background:**

India is the largest milk producer globally, with the largest proportion of cattle milk production coming from smallholder farms with an average herd size of less than two milking cows. These cows are mainly undefined multi-generation crosses between exotic dairy breeds and indigenous Indian cattle, with no performance or pedigree recording. Therefore, implementing genetic improvement based on genetic evaluation has not yet been possible. We present the first results from a large smallholder performance recording program in India, using single nucleotide polymorphism (SNP) genotypes to estimate genetic parameters for monthly test-day (TD) milk records and to obtain and validate genomic estimated breeding values (GEBV).

**Results:**

The average TD milk yield under the high, medium, and low production environments were 9.64, 6.88, and 4.61 kg, respectively. In the high production environment, the usual profile of a lactation curve was evident, whereas it was less evident in low and medium production environments. There was a clear trend of an increasing milk yield with an increasing Holstein Friesian (HF) proportion in the high production environment, but no increase above intermediate grades in the medium and low production environments. Trends for Jersey were small but yield estimates had a higher standard error than HF. Heritability estimates for TD yield across the lactation ranged from 0.193 to 0.250, with an average of 0.230. The additive genetic correlations between TD yield at different times in lactation were high, ranging from 0.846 to 0.998. The accuracy of phenotypic validation of GEBV from the method that is believed to be the least biased was 0.420, which was very similar to the accuracy obtained from the average prediction error variance of the GEBV.

**Conclusions:**

The results indicate strong potential for genomic selection to improve milk production of smallholder crossbred cows in India. The performance of cows with different breed compositions can be determined in different Indian environments, which makes it possible to provide better advice to smallholder farmers on optimum breed composition for their environment.

## Background

India is the world’s largest producer of milk, producing 198.40 $\times$ 10^9^ kg milk per annum, increasing by approximately 5.68% per annum [1]. Some 49% of milk is produced by buffaloes and 49% by cattle, of which 57% come from crossbreds between indigenous *Bos indicus* and exotic *Bos taurus* dairy breeds, with an average yield of 8.09 kg/day [1], and an average herd size of less than two cows. Crossbred cows are used in India to combine the production potential of exotic dairy breeds with the adaptation to difficult environments of indigenous *Bos indicus* cattle, and to use the well-documented heterosis in such crossbreds in tropical environments [2–5].

Smallholder indigenous $\times$ exotic crossbred dairy cattle in India and elsewhere form populations of continuous breed composition, with the proportion of exotic breed origin ranging from almost 0 to almost 100%, and with the vast majority of replacement heifers being calves of crossbred cows mated to crossbred or purebred bulls. Implementing genetic improvement strategies in smallholder crossbred populations faces many challenges, the principal being the lack of performance or pedigree recording and the uncertainty about what accuracy can be achieved when herd sizes are so small and production environments vary widely between farms and over time [6]. An obstacle to initiating genetic improvement in such populations is funding and action-fatigue because of the long lag time between when performance recording is initiated and when the amount of pedigree information accumulated is sufficient to generate breeding values. However, the lag can be reduced from many years to about a year, if animals that are performance-recorded are genotyped with a medium or high-density single nucleotide polymorphism (SNP) chip and the information is used to generate a genomic relationship matrix (GRM). This approach is being applied in East Africa, where a pilot program of smallholder recording of about 1200 cows in Kenya achieved heritabilities of 0.05 to 0.27 and accuracies of genomic estimated breeding values (GEBV) from 0.28 to 0.44 depending on the method [7, 8].

BAIF Development Research Foundation (BAIF—http://www.baif.org.in) is a large NGO that delivers livestock development services to over 2.94 million farm households in India. BAIF produces more than 13.5 million frozen semen doses annually for delivery to smallholder dairy cattle and buffaloes, and performs about 5 million artificial inseminations per year. BAIF has initiated a large smallholder dairy recording program across six states in India, where recorded crossbred cows as well as crossbred and pure-exotic bulls in the artificial insemination (AI) stud are genotyped to generate estimates of breed composition and GEBV. Here, we report the first genetic analyses of this population, based on 73,968 test-day (TD) milk records of 3842 cows with phenotypes and genotypes. Our results demonstrate useful levels of heritability and validated accuracies of GEBV. We also report estimates of in-situ performance of different classes of crossbred animals across varying production environments.

## Methods

### Milk data

The data analysed here originate from the Enhanced Genetic Gains (EGP) program of BAIF. Phenotype and associated data are from cows in smallholder herds ranging from 1 to 43 cows in size, collected in the States of Bihar, Jharkhand, Maharashtra, Odisha, and Punjab Uttar Pradesh between February 2016 and May 2019. All cows were crossbreds, of unknown breed composition, between exotic *Bos taurus* dairy breeds and local indigenous *Bos indicus* cattle. In the areas that were sampled, the only exotic dairy breeds known to have been used in the past 50 years of crossbreeding are Holstein/Friesian (HF) and Jersey (JR). The available data included TD milk yields, calving date, parity, date of birth, age, herd, village, CDC (Cattle Development Centre, i.e. a local dairy service centre that covers 8 to 10 villages, under which AI delivery and recording are organised), district, and State.

A wide variety of quality control (QC) measures were explored, resulting in the following sequential QC steps being applied to produce data that are suitable for analysis. The first step was to ensure that we had a sufficient amount of data on each cow to be able to perform basic tests of reliability of the data. In the following steps, relatively high limits were imposed, which reflect the high within- and between-cow variability in data from smallholder systems compared to intensive dairy systems and the desire to avoid extreme outliers:

1. Retain cows with four or more monthly TD milk yield between 8 and 340 days after calving, in at least one lactation.
2. Remove cows with an average TD milk yield greater than 4 standard deviations (SD) above the population average.
3. Remove cows with SD of TD milk yield greater than 4 SD above the population average.
4. Remove cows with a coefficient of variation (CV) of TD milk yield higher than 0.8.
5. Based on an initial statistical analysis (see below), remove individual records for which the residual is outside the − 4 to 4 kg/day range, and remove cows with a SD of residuals greater than 2.5 kg/day.
6. Data for CDC with less than 10 cows in a given State were combined to form a dummy CDC. Any dummy CDC with less than 10 cows was deleted.

In total, 8934 crossbred cows with four or more monthly milk records between 8 and 340 days after calving were retained. After applying all QC steps, 8563 crossbred cows with 149,508 TD records in 4277 herds, within 728 villages, 87 CDC, and 30 districts, remained.

The number of cows per herd ranged from 1 to 43 (Table 1), and 23% of the cows were in herds of one cow and 73% of the cows were in herds with three or less cows. The number of cows per CDC ranged from 10 to 349.

**Table 1:** Number of herds and crossbred cows in each herd size

Herd size	1	2	3	4	5	6	7	8	9	10	11	12–43
Herds	2005	1294	547	239	78	43	17	13	11	9	5	16
Cows	2005	2588	1641	956	390	258	119	104	99	90	55	258

In earlier statistical analyses (results not shown), the effects of district, CDC, village, herd and animal were fitted as random variables, along with a series of fixed effects. District and village explained very little variation and were not included in subsequent models. Due to the relatively large number of cows in each CDC, CDC were then treated as fixed effects and their interaction with lactation curves was explored.

### Genotypes

In total, 4938 crossbred cows were genotyped, of which 640 were genotyped with the Illumina 777k BovineHD BeadChip (Illumina Inc., San Diego, CA, USA) and 4298 genotyped with the GeneSeek Genomic Profiler (GGP) Bovine 50K BeadChip (Neogen GeneSeek Operations, Lincoln, NE, USA). Furthermore, genotype data were available for 733 animals (cows and bulls) from the BAIF bull stud herd, 518 of which were genotyped with the Illumina BovineSNP50 array and the remaining genotyped with the GGP Bovine 50K array. The stud animals consisted of purebred HF, purebred JR, HF $\times$ indigenous crosses and JR $\times$ indigenous crosses. Autosomal SNPs with a GC (GenCall) score higher than 0.15, with a call rate higher than 0.9 and a MAF higher than 0.01 were retained. Samples with a call rate lower than 0.9 were excluded. Duplicate samples were identified based on a correlation between genotypes higher than 0.98. All duplicate cow samples were removed because it was not possible to assign the correct milk records to the genotypes. One copy of each set of duplicates among stud animals was retained, because stud animals had no milk records and thus their inclusion is not a source of errors in the genetic analyses.

Following the application of the genotype QC, all animals genotyped with the 50 K chip were then imputed to high-density (HD) using a reference set of 2961 animals with real HD genotypes. The reference set for imputation included 623 crossbred cows from our dataset, 686 Indian indigenous animals, 968 pure Holsteins and 684 pure Jerseys sourced from the Bovine HapMap [9]. The Indian indigenous reference and HapMap datasets were genotyped on the Illumina 777k BovineHD BeadChip and were quality-controlled. Imputation was performed using the FImpute software [10] and resulted in 737,073 SNP genotypes across the 29 *Bos taurus* autosomes. After applying the genotype QC and retaining the cows that passed the phenotype QC, 3842 crossbred cows with genotype and milk records and 661 stud animals without milk records were available for the genetic analyses.

### Phenotypic and genetic analyses

The analyses of TD milk data were performed in a two-stage process. In the first stage, TD records were corrected for fixed effects using a fixed effect linear model (Model 1) and a mixed linear model (Model 2) to obtain adjusted TD records. Then, the adjusted records were used in the second stage to obtain estimates of genetic parameters and GEBV using a random regression (RR) model. A two-step analysis was used because, among the 8563 cows with TD records that passed QC, only 3842 had genotypes. Accounting for fixed effects is substantially more accurate when including all the cows with phenotypes.

Three methods for adjusting TD records for non-genetic effects were investigated. The first method adjusted TD records for fixed effects using Model 1 as follows:

1 $$\mathbf{y}=\mathbf{X}\mathbf{b}+\mathbf{e},$$

where $\mathbf{y}$ is the vector of TD records and $\mathbf{b}$ is the vector of fixed effects, $\mathbf{X}$ is the design matrix that links the fixed effects to the TD records, and $\mathbf{e}$ is the vector of residuals. Fixed effects included lactation number, year-month (2016–09 to 2019–07), CDC, interaction of year-month by CDC, average lactation curve modelled by a 3rd-order Legendre polynomial (LP), lactation curves for each parity modelled by the 3rd order LP, and lactation curves for each CDC modelled by the 3rd order LP. The 3rd order LP was found to be a better fit than the 1st and 2nd order LP for modelling the observed high variability of curvature.

The other two methods for obtaining adjusted TD records involved fitting an animal repeatability model (Model 2) as follows:

2 $$\mathbf{y}=\mathbf{X}\mathbf{b}+{\mathbf{Z}}_{1}\mathbf{a}+{\mathbf{Z}}_{2}\mathbf{h}+\mathbf{e}.$$

Model 2 is the same as Model 1 with the addition of random effects of animal and herd under a repeatability model (i.e. individual animal and herd lactation curves are not fitted), where $\mathbf{a}$ and $\mathbf{h}$ are the vectors of random animal and herd effects, respectively, and ${\mathbf{Z}}_{1}$ and ${\mathbf{Z}}_{2}$ are the design matrices that link the random animal and herd effects to the TD records, respectively, assuming that all the animals are unrelated. The two methods for obtaining adjusted TD records from Model 2 were:

Model 2-M1: The estimated animal effects ($\widehat{\mathbf{a}}$) were added to the corresponding herd effects ($\widehat{\mathbf{h}}$) and residual effects ($\widehat{\mathbf{e}}$) to obtain the adjusted TD records. In this case, milk records were adjusted only for the fixed effects in the mixed model.

Model 2-M2: The estimated animal effects ($\widehat{\mathbf{a}}$) were added to the corresponding residual effects ($\widehat{\mathbf{e}}$) to obtain the adjusted TD records. In this case, milk records were adjusted for fixed effects plus the random herd effect in the mixed model.

### Estimation of genetic parameters and GEBV

Genetic parameters and GEBV were obtained using an RR model. The analysis included 73,968 TD records on 3842 crossbred cows that passed both the phenotype and genotype QC, plus 661 bull stud animals that passed the genotype QC. The analysis also included 24,063 TD records for 1575 crossbred cows that passed the phenotype QC and do not have genotypes but are in the same herds as the cows with genotypes plus phenotypes. This was done to improve the accuracy of the estimation of herd versus animal effects in herds with few cows.

The RR model that was fitted to the adjusted TD records obtained from Model 1 and Model 2-M1 includes the random effects of additive genetic, permanent environment (PE), and herd:

3 $${\mathbf{y}}^{\mathbf{*}}={\mathbf{Z}}_{1}\mathbf{a}+{\mathbf{Z}}_{2}\mathbf{p}+{\mathbf{Z}}_{3}\mathbf{h}+\mathbf{e},$$

where ${\mathbf{y}}^{\mathbf{*}}$ are the adjusted TD records, $\mathbf{a}$ contains the $m+1$ additive genetic regression coefficients for each animal, $\mathbf{p}$ contains the $m+1$ PE regression coefficients for each animal, $\mathbf{h}$ contains $m +1$ random regression coefficients for each herd, $m$ is the order of LP, $\mathbf{e}$ contains the residuals, and ${\mathbf{Z}}_{1}$, ${\mathbf{Z}}_{2}$, and ${\mathbf{Z}}_{3}$ are the incidence matrices of additive genetic, PE, and herd random regression coefficients, respectively. Days in milk (DIM) that ranged from 8 to 340 were used to generate LP. Residual variance was assumed to be homogenous for TD records within, but heterogeneous between the eight lactation period classes: 8–49, 50–91, 92–133, 134–175, 176–217, 218–259, 260–301, and 302–340 days post-calving. It was assumed that:

$$var\left( {\begin{array}{*{20}c} {\begin{array}{*{20}c} {\mathbf{a}} \\ {\mathbf{p}} \\ \end{array} } \\ {\mathbf{h}} \\ {\mathbf{e}} \\ \end{array} } \right) = \left( {\begin{array}{*{20}c} {{\mathbf{G}} \otimes {\mathbf{K}}_{{\mathbf{a}}} } & 0 & 0 \\ 0 & {{\mathbf{I}} \otimes {\mathbf{K}}_{{\mathbf{p}}} } & 0 \\ {\begin{array}{*{20}c} 0 \\ 0 \\ \end{array} } & {\begin{array}{*{20}c} 0 \\ 0 \\ \end{array} } & {\begin{array}{*{20}c} {{\mathbf{I}} \otimes {\mathbf{K}}_{{\mathbf{h}}} } \\ 0 \\ \end{array} } \\ \end{array} \begin{array}{*{20}c} {\begin{array}{*{20}c} 0 \\ 0 \\ \end{array} } \\ {\begin{array}{*{20}c} 0 \\ {\mathbf{R}} \\ \end{array} } \\ \end{array} } \right)$$

where ${\mathbf{K}}_{\mathbf{a}}$, ${\mathbf{K}}_{\mathbf{p}}$, and ${\mathbf{K}}_{\mathbf{h}}$ are the (co)variance matrices of the additive genetic, PE and herd regression coefficients, respectively, $\mathbf{R}$ is a diagonal matrix of residual variances, $\mathbf{G}$ is the genomic relationship matrix constructed using the first method of VanRaden [11]. Animals without genotypes were assumed to be unrelated to all other animals with diagonal elements of 1 in $\mathbf{G}$ (the average of the diagonals of the GRM built from the genotyped cows is 1). $\otimes$ is the direct product operation and $\mathbf{I}$ is an identity matrix with dimensions equal to the number of levels of effects. Variance components and the elements of ${\mathbf{K}}_{\mathbf{a}}$, ${\mathbf{K}}_{\mathbf{p}}$, ${\mathbf{K}}_{\mathbf{h}}$, and $\mathbf{R}$ were estimated using an Average Information Restricted Maximum Likelihood (AI-REML) procedure in the Wombat software [12]. First order LP (${\alpha }_{0}$ and ${\alpha }_{1}$) were fitted to the random additive genetic, PE and herd effects. Preliminary analyses indicated that fitting an LP higher than the first order had almost no effect on model fit, thus only the results from the first order LP are presented.

The variance–covariance matrices of the additive genetic (${\mathbf{V}}_{\mathrm{a}}$), PE (${\mathbf{V}}_{\mathrm{pe}}$) and herd (${\mathbf{V}}_{\mathrm{h}}$) components at the eight DIM (28, 70, 112, 154, 197, 238, 280, and 321 days), representing the midpoint of the eight TD classes used to group the heterogeneous residual variances, were calculated as:

$$\mathbf{V}= {\varvec{\upphi}}\mathbf{K}{{\varvec{\upphi}}}^{\mathrm{^{\prime}}},$$

where $\mathbf{K}$ is the (co)variance matrix of regression coefficients (${\mathbf{K}}_{\mathbf{a}}$, ${\mathbf{K}}_{\mathbf{p}}$, or ${\mathbf{K}}_{\mathbf{h}}$), ${\varvec{\upphi}}$ is $8\times 2$ matrix of LP (intercept and slope) on selected DIM. In most of the published literature, herd and its interactions such as herd-year-season (HYS) is fitted as a fixed effect and hence herd does not contribute to the estimate of phenotypic variance used to estimate the heritability. Thus, to be able to compare our results with most of those in the literature, we estimated the heritability (${h}_{i}^{2}$) at TD $i$ as:

$${h}_{i}^{2}= \frac{{\sigma }_{a(i)}^{2}}{{\sigma }_{a(i)}^{2}+{\sigma }_{pe(i)}^{2}+{\sigma }_{e(i)}^{2}},$$

where ${\sigma }_{a(i)}^{2}$, ${\sigma }_{pe(i)}^{2}$, and ${\sigma }_{e(i)}^{2}$ are the additive genetic, PE, and residual variances at TD $i$, respectively, $i$ is one of the selected eight TD records. The additive genetic correlations (${r}_{a(i,j)}$) and PE correlations (${r}_{pe(i,j)}$) between TD $i$ and $j$ were obtained as:

$${{{r}}}_{{{a}}({{i}},{{j}})}=\frac{{{{\sigma}}}_{{{a}}({{i}},{{j}})}}{\sqrt{{{{\sigma}}}_{{{a}}({{i}})}^{2}.{{{\sigma}}}_{{{a}}({{j}})}^{2}}},$$

$${{{r}}}_{{{p}}{{e}}({{i}},{{j}})}=\frac{{{{\sigma}}}_{{{p}}{{e}}({{i}},{{j}})}}{\sqrt{{{{\sigma}}}_{{{p}}{{e}}({{i}})}^{2}.{{{\sigma}}}_{{{p}}{{e}}({{j}})}^{2}}},$$

where ${\sigma }_{a(i,j)}$ and ${\sigma }_{pe(i,j)}$ are the additive genetic and PE covariance between TD $i$ and $j$, respectively. In our data, the average number of TD milk records per cow over the lactation was 7.8. For simplicity, we assumed that each cow had one TD record in each heterogeneous residual class. Then, the $8\times 8$ (co)variance matrices (${\mathbf{V}}_{\mathrm{a}}$ and ${\mathbf{V}}_{\mathrm{pe}}$), as well as the $8\times 8$ diagonal residual matrix (${\mathbf{V}}_{\mathrm{e}}$) were used to calculate the heritability (${h}_{\mathrm{W}}^{2}$) for the whole lactation period for a typical cow as:

$${{{h}}}_{\text{W}}^{2}=\frac{\sum {\mathbf{V}}_{\text{a}}}{\sum {\mathbf{V}}_{\text{a}}+\sum {\mathbf{V}}_{\text{p}\text{e}}+\sum {\mathbf{V}}_{\text{e}}}.$$

For the adjusted TD records obtained from Model2-M2, the following RR model (Model 4) was fitted:

4 $${\mathbf{y}}^{\mathbf{*}}={\mathbf{Z}}_{1}\mathbf{a}+{\mathbf{Z}}_{2}\mathbf{p}+\mathbf{e}.$$

Model 4 is the same as Model 3 except that the random effect of herd was not included since the data had been adjusted for herd in the pre-correction step.

### Breed composition and environmental classes

An estimate of the environment that a given cow experiences was obtained as the sum of the estimates of the effects of the CDC and herd of that cow, obtained from Model 2. These estimates of the cow’s environment were then ranked and grouped into the top, middle, and bottom one third, to create a fixed effect for environment (ENV) with three levels, high, medium and low production environments.

Breed composition for each cow was estimated from SNP genotypes using a supervised Admixture analysis [13] with four ancestral populations: Holstein, Friesian, Jersey and Indian *Bos indicus* indigenous. The Indian ancestral reference population consisted of 94 animals selected from the Indian indigenous reference animals, which were selected to represent the least related animals within a breed, with the highest average kinship with unselected animals [14]. The animals selected were in proportion with the total number of animals available for each breed. The exotic reference population consisted of 21 pure Holstein and 21 Jersey animals from the bovine HapMap [9], and 21 Friesian animals from the Scottish Rural University College (SRUC).

The estimates of breed composition were used to group cows into four breed composition classes (0–25%, 25–50%, 50–75%, and 75–100%) based on their proportion of HF ancestry and separately for their proportion of JR ancestry, from which resulted 10 possible combinations of HF and JR classes. These 10 possible combinations are four breed combinations between the HF class (0–25%) and JR classes (0–25%, 25–50%, 50–75%, and 75–100%); three breed combinations between the HF class (25–50%) and JR classes (0–25%, 25–50%, and 50–75%); two breed combinations between the HF class (50–75%) and JR classes (0–25% and 25–50%), and one breed combination between the HF class (75–100%) and JR class (0–25%). Cows without genotypes were allocated to either an HF cross (HFx) or a JR cross (JRx), based on their assignment by enumerators in the field using the cow's appearance and farmer’s information. This resulted in 12 classes of breed composition. The interaction between breed composition and environment was examined by fitting an effect with 36 levels for breed composition (12 classes) by environment (3 classes). This effect was included either in the phenotype analysis used to generate adjusted TD yields for genetic analysis, or in the genetic analysis.

### Validation of GEBV for crossbred performance

The validation of GEBV in this dataset was performed on the genotyped cows, rather than the bulls, because SNP-based parentage assignment matched 25% of the genotyped cows to a genotyped bull, and most bulls that had progeny in the dataset had very few progeny (see "Results" section). Validation was performed for the GEBV for ${\alpha }_{0}$. No validation for ${\alpha }_{1}$ was attempted because the genetic variation of ${\alpha }_{1}$ was too low to yield a meaningful validation. The validation for ${\alpha }_{0}$ was performed by identifying a validation set of cows, whose phenotypes were then masked in the genetic evaluation. Ten or 11 non-overlapping sets of validation cows were identified and the GEBV were generated separately for each validation set. Then, the validation GEBV were combined across all validation sets and the correlations between the GEBV and alternative measures of TD performance of each cow were estimated (see below). Cross-validations were performed as follows: (A) by random sampling of 10 non-overlapping subsets of cows from the whole dataset, and (B) by random sampling of 11 non-overlapping subsets of CDC from the data, and allocating all cows in the selected CDC to the validation set (11 sets were used to approximately balance the size of subsets allowing for a large variation in the number of cows per CDC, see Table 2). Of these two methods, we expect that method B will yield the least biased estimates of accuracy because there is minimal sharing of environmental effects on phenotype between the validation set and the cows with phenotypes used to generate the GEBV. Accuracies from method A are expected to be biased upward due to significant sharing of phenotype effects between the cows in the validation set and those with phenotypes used to generate the GEBV.

**Table 2:** Number of cows in each CDC validation subset

Validation subset	1	2	3	4	5	6	7	8	9	10	11
Cows	396	400	294	326	370	321	346	380	382	364	250
CDC	6	9	7	5	11	7	4	8	8	11	4

*CDC* Cattle Development Centre

The accuracy of prediction was estimated as the correlation between the GEBV for ${\alpha }_{0}$ and cow TD phenotypes, divided by the $\sqrt{{h}^{2}}$ of ${\alpha }_{0}$. Three alternative TD phenotypes were used: (1) the cows’ average unadjusted TD record; (2) the cows’ average adjusted TD record; and (3) the cows’ average TD record corrected for CDC. The heritability of ${\alpha }_{0}$ in this context, for the whole lactation period, was 0.217, calculated as the proportion of the additive genetic variance relative to the total phenotypic variance (i.e. including herd variance). The coefficient of the regression of target phenotypes on GEBV for ${\alpha }_{0}\times$ 0.7071 was calculated to evaluate the bias of predictions, where 0.7071 = $0.5\times \sqrt{2}$ is the scaling factor to convert ${\alpha }_{0}$ to the same scale as TD milk. For comparison with the estimates of accuracy from the validation analyses, the accuracy of GEBV was also estimated from the GEBV analyses as: $\sqrt{1-\frac{PEV}{{\sigma }_{a}^{2}}}$, where $PEV$ is the prediction error variance estimated from the mixed model equations and ${\sigma }_{a}^{2}$ is the additive genetic variance.

## Results

### Phenotype analysis

The inclusion of fixed effects in the Step 1 analyses explained 52% of the phenotypic variance for Model 1 and 67% of the variance for Model 2. Least square means of CDC effects ranged from 2.97 to 13.36 kg/day, and for year-month ranged from 6.58 to 7.10 kg/day. CDC effects occur within States and the large range in CDC effects reflects large differences in production environments between States as well as regional differences within States. For example, the average TD yield for the Odisha State with the lowest yield was 3.76 kg/day, while that for the Maharashtra State with the highest yield was 8.39 kg/day.

### Model selection

The estimates of (co)variances and correlations between the first (${\alpha }_{0}$) and second (${\alpha }_{1}$) random regression (RR) coefficients obtained from the RR models (Models 3 and 4) using different methods to adjust TD milk yield are in Table 3. The first RR coefficient (${\alpha }_{0}$) corresponds to the mean milk yield and the second RR coefficient (${\alpha }_{1}$) corresponds to the persistency of yield (the rate of decline in milk yield from the first DIM in the analysis; i.e. day 8 of lactation in our analysis). In Model 3, the estimate of the additive genetic variance of ${\alpha }_{0}$ was about 14% higher when using the adjusted TD from Model 2-M1 (1.256) compared to Model 1 (1.097), whereas the estimates of the additive genetic variance of ${\alpha }_{1}$ were very similar in both cases. However, the average of the heterogeneous residual variance was smaller when using adjusted TD from Model 2-M1 (1.073) compared to Model 1 (1.169). For Model 4, in which TD records were adjusted for fixed effects and random herd effects from Model 2-M2, the estimated additive genetic variance of ${\alpha }_{0}$ was significantly reduced (0.470) compared to that obtained with Model 3. For Model 3, the contribution of ${\alpha }_{0}$ to the additive genetic effect of individual TD yields was much greater than that of ${\alpha }_{1}$, the first eigenvalue of the additive genetic co-variance matrix explaining ~ 90 to ~ 97% of the additive genetic variation. In both Models 3 and 4, the correlations between ${\alpha }_{0}$ and ${\alpha }_{1}$ were negative and moderate, ranging from − 0.21 to − 0.36, indicating that higher yields are associated with less persistent lactations.

**Table 3:** Estimates of variances (diagonals), co-variances (above the diagonal), and correlations (below the diagonal) between the random regression coefficients, along with the two eigenvalues (λ) and the relative proportions of variance that they explain (%)

	Additive genetic		Herd		Permanent environment		Average residual^*^
	${\alpha }_{0}$	${\alpha }_{1}$	${\alpha }_{0}$	${\alpha }_{1}$	${\alpha }_{0}$	${\alpha }_{1}$	
*Model 3 using adjusted TD from Model 1*							
${\alpha }_{0}$	1.097 (0.210)	− 0.093 (0.062)	3.584 (0.171)	− 0.349 (0.049)	2.908 (0.194)	− 0.357 (0.064)	1.169
${\alpha }_{1}$	− 0.345 (0.211)	0.067 (0.036)	− 0.355 (0.04)	0.270 (0.026)	− 0.228 (0.038)	0.842 (0.043)	
λ (%)	1.11 (94.98)	0.06 (5.02)	3.62 (93.93)	0.23 (6.07)	2.97 (79.15)	0.78 (20.85)	
*Model 3 using adjusted TD from Model 2-M1*							
${\alpha }_{0}$	1.256 (0.227)	− 0.105 (0.063)	4.063 (0.185)	− 0.357 (0.048)	2.854 (0.204)	− 0.331 (0.063)	1.072
${\alpha }_{1}$	− 0.357 (0.195)	0.069 (0.033)	− 0.364 (0.043)	0.237 (0.023)	− 0.229 (0.040)	0.734 (0.038)	
λ (%)	1.27 (95.50)	0.06 (4.50)	4.10 (95.26)	0.20(4.74)	2.90 (80.95)	0.68 (19.05)	
*Model 4 using adjusted TD from Model 2-M2*							
${\alpha }_{0}$	0.470 (0.105)	− 0.094 (0.048)	–	–	2.321 (0.102)	− 0.299 (0.048)	1.073
${\alpha }_{1}$	− 0.346 (0.158)	0.158 (0.042)	–	–	− 0.210 (0.031)	0.867 (0.043)	
λ (%)	0.50 (78.98)	0.13 (21.02)	–	–	2.38 (74.64)	0.81 (25.36)	
Model 3: BC*ENV in the genetic analysis							
${\alpha }_{0}$	1.562 (0.233)	− 0.149 (0.066)	2.875 (0.151)	− 0.303 (0.043)	2.575 (0.200)	− 0.289 (0.063)	1.072
${\alpha }_{1}$	− 0.459 (0.184)	0.068 (0.033)	− 0.369 (0.046)	0.235 (0.023)	− 0.211 (0.043)	0.734 (0.038)	
λ (%)	1.58 (96.75)	0.05 (3.25)	2.91 (93.54)	0.20 (6.46)	2.62 (79.16)	0.69 (20.84)	
Model 3: BC*ENV in the phenotype analysis							
${\alpha }_{0}$	0.642 (0.170)	− 0.091 (0.057)	1.010 (0.081)	− 0.088 (0.031)	3.034 (0.165)	− 0.293 (0.058)	1.073
${\alpha }_{1}$	− 0.388 (0.214)	0.085 (0.034)	− 0.182 (0.062)	0.228 (0.023)	− 0.199 (0.037)	0.719 (0.038)	
λ (%)	0.66 (90.30)	0.07 (9.70)	1.02 (82.33)	0.22 (17.67)	3.07 (81.83)	0.68 (18.17)	

^*^Average of residual variance estimates for the eight lactation period classes: 8–49, 50–91, 92–133, 134–175, 176–217, 218–259, 260–301, and 302–340 days of lactation. The estimates of residual variances are for test-day milk yield in kg/dayay, whereas the genetic, PE, and herd variances are on the LP scale. The standard error of the estimates is given in brackets

### Accounting for breed composition and its interaction with environment

**Fig. 1:**
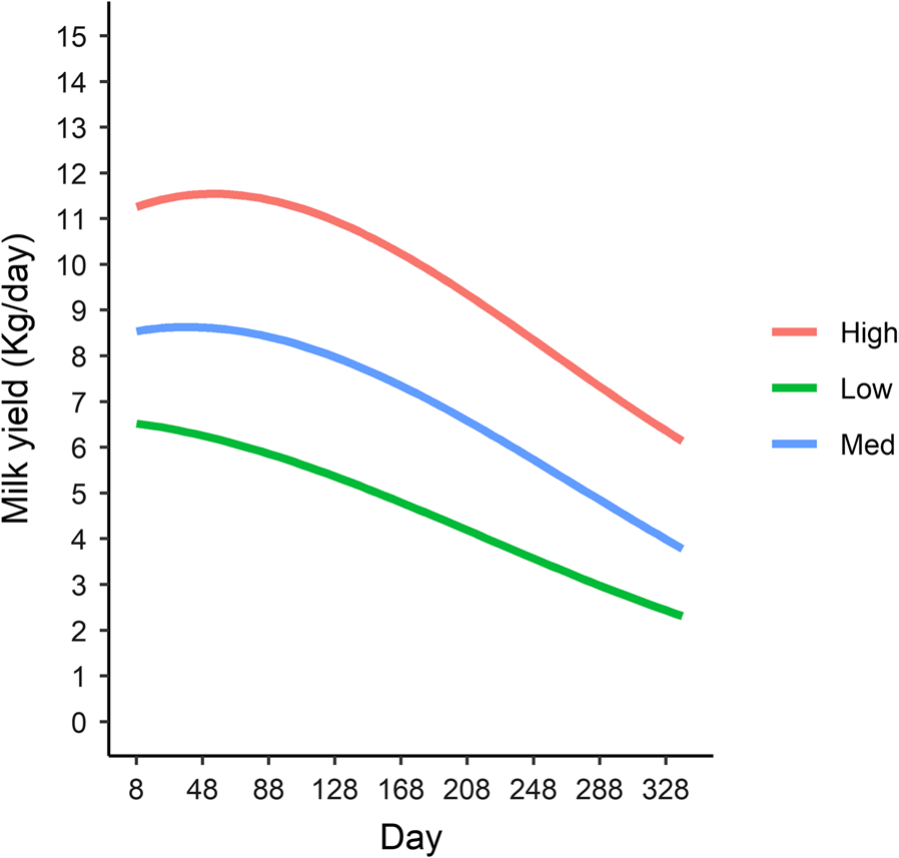
Lactation curves in high, medium, and low production environments

Lactation curves for high, medium, and low production environments obtained from Model 2 are shown in Fig. 1. The predicted average daily milk yield under the three environments (high, medium, and low) were 9.64, 6.88, and 4.61 kg/day, respectively. The lactation curve of cows in the high production environment shows a shallow peak during early lactation, which was barely present in the medium production environment and not present in the low production environment.

**Fig. 2:**
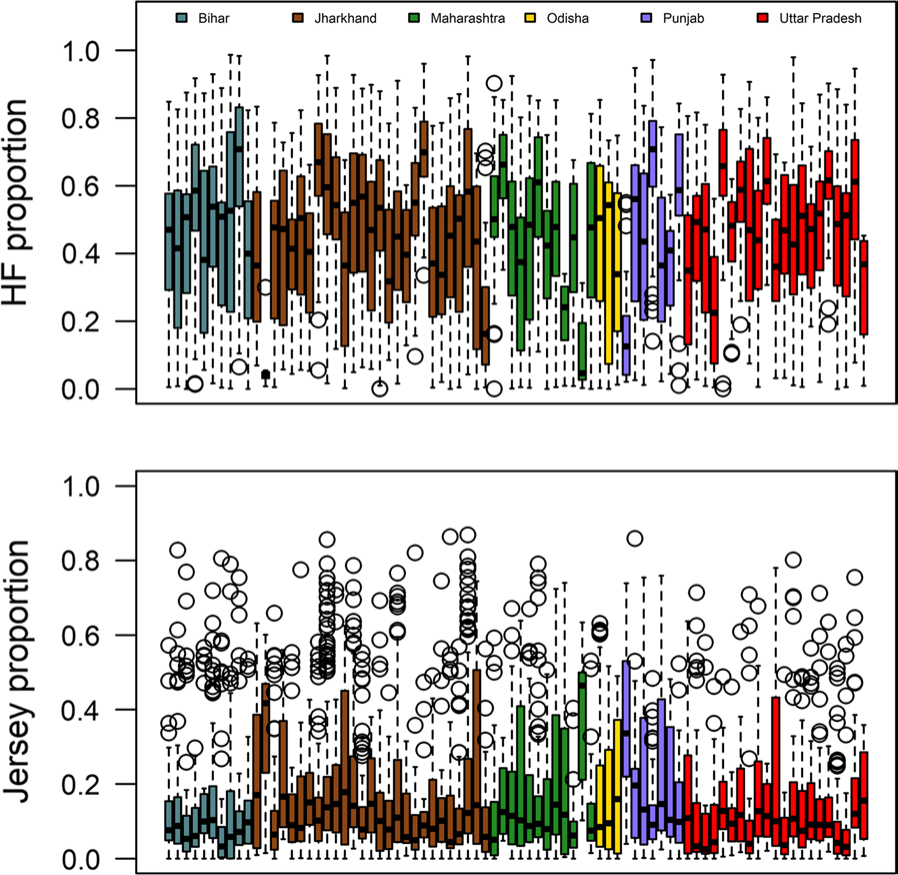
Distribution of Holstein/Friesian (HF) and Jersey (JR) proportions for the genotyped animals for each state and CDC. Each bar represents a CDC and each state is represented by a different colour

**Fig. 3:**
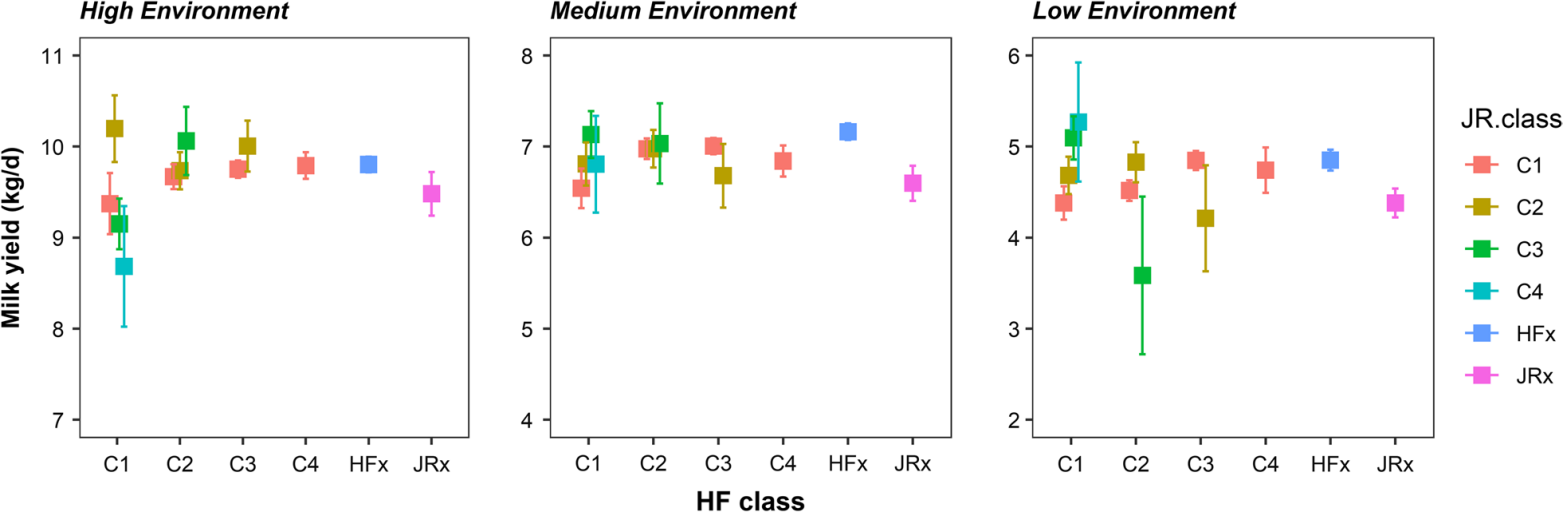
Estimated effects of breed composition by environment when fitted in the genetic model. Error bars represent the standard error of the estimates. Within each environment, the average breed composition estimate for that environment was subtracted from each of the breed composition estimates and the average milk yield for that environment was then added. C1 to C4 are the four classes of HF or JR breed composition as estimated from SNP genotypes, and HFx and JRx are ungenotyped animals classified as HF or JR crosses in the field

**Fig. 4:**
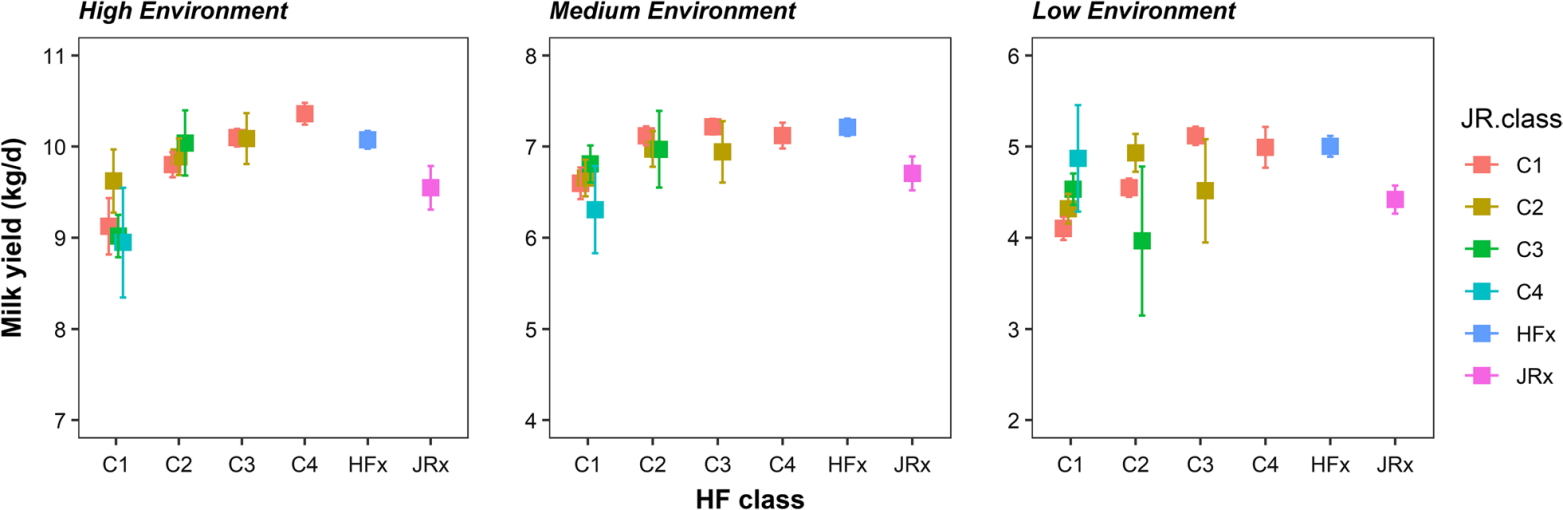
Estimated least-square (LS) means of breed composition by environment when fitted in the pre-adjustment phenotype model. Error bars represent the standard error of the estimates. Breed classes identical to Fig. 3

Breed composition varied markedly across CDC and across States (Fig. 2). This means that adjustment of TD records for the fixed effect of CDC, when breed composition is not included in the model, will absorb some of the breed composition effects, which potentially leads to underestimate the breed composition effects when they are included in the genetic analysis. This is seen in the results obtained when breed composition is fitted in the genetic analysis (Fig. 3) compared to those when it is fitted in the phenotype analysis (Fig. 4). Because the derivation of ENV is based on estimates of the herd random effect plus the CDC fixed effect, when using the same adjusted TD data, fitting BC $\times$ ENV in the genetic analysis substantially reduces the herd variance to 2.875, compared to 4.063 when BC $\times$ ENV is not fitted in the genetic analysis (Table 3). For the same comparison, fitting BC $\times$ ENV in the genetic analysis increased the estimated additive genetic variance of ${\alpha }_{0}$ from 1.256 to 1.562 compared to not fitting BC $\times$ ENV, while the PE variance decreased slightly from 2.853 to 2.575, and the residual variance was hardly affected.

As expected, fitting BC $\times$ ENV in the phenotype analysis absorbed a large part of the CDC and herd variances and caused a substantial drop in the animal variance (result not shown). Consequently, in the subsequent genetic analysis (last row of Table 3), there was a 49% drop in the estimated additive genetic variance of ${\alpha }_{0}$ (from 1.256 to 0.642) and a 75% drop in the estimated herd variance of ${\alpha }_{0}$ (from 4.063 to 1.01). However, the estimate of PE variance was not much affected, going from 2.854 to 3.034.

In the high production environment, there was a strong upward trend in yield as HF ancestry increased, when BC $\times$ ENV effects were estimated in the phenotype analysis (Fig. 4) and a substantially smaller trend when included in the genetic analysis (Fig. 3). Trends were less clear in the medium and low production environments, although the cows with more HF blood still tended to have higher yields than those with less HF blood. This population had a lower average and less variable proportion of Jersey breed compared to the proportion of HF breed, leading to larger SE of the estimates of Jersey breed composition classes. If the estimates with very high SE were ignored, there was a general trend for higher grade Jersey classes to yield more than the lower grade classes in the medium and low production environments, although the differences are small with regards to the errors of estimation (Fig. 3).

### Variance components and heritability estimates for DIM

**Fig. 5:**
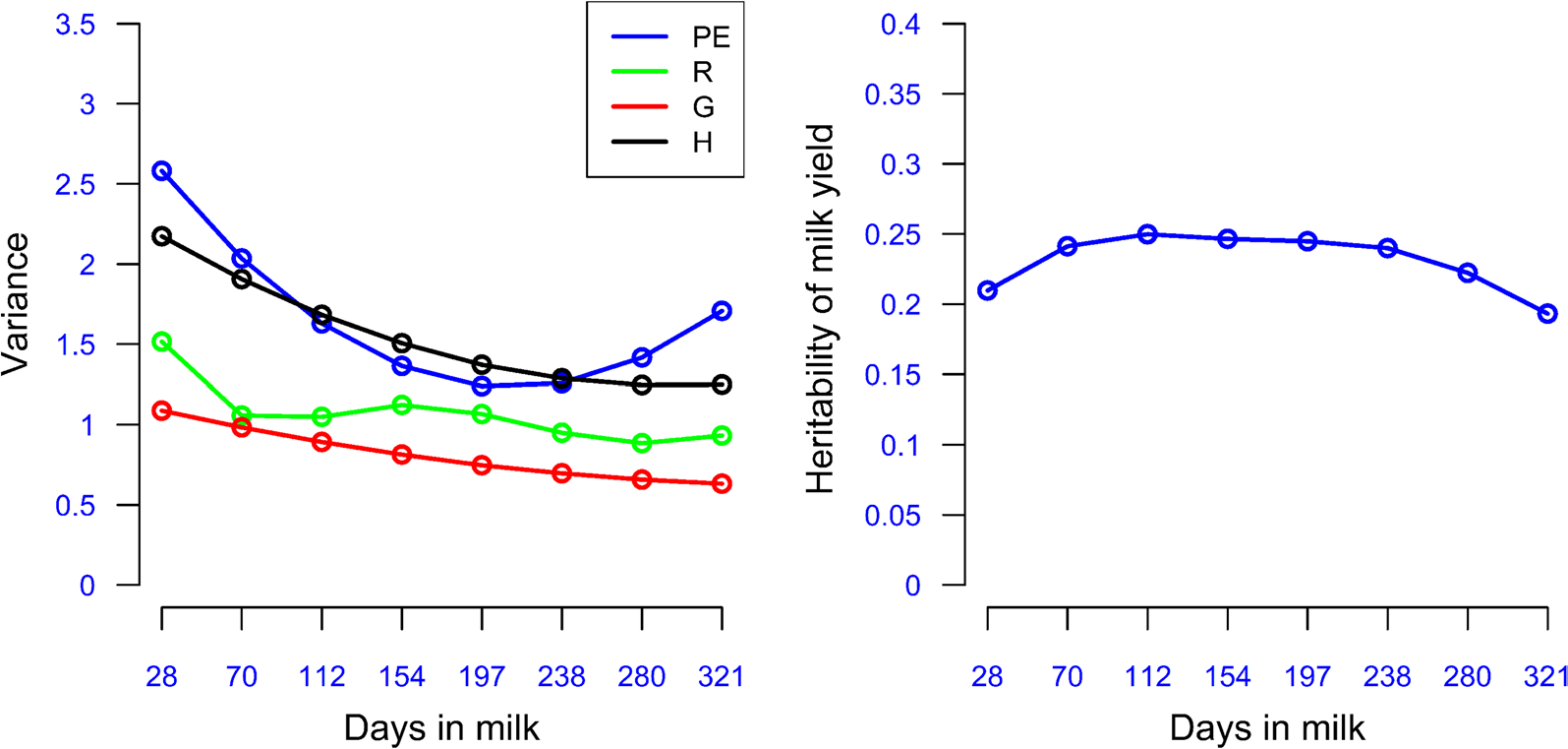
Left plot: Additive genetic (G), herd (H), permanent environmental (PE), and residual (R) variance estimates; and right plot: heritability estimates derived from the left plot

Genetic parameters for TD yields across the lactation were estimated using Model 3, in which the fixed effect of BC $\times$ ENV was fitted along with the random additive genetic, herd, and PE effects. The estimates of additive genetic, herd, and PE variances for the eight DIM (28, 70, 112, 154, 197, 238, 280, and 321), the midpoints of the eight DIM classes used to group the heterogeneous residual variances, are shown in Fig. 5. The additive genetic variance steadily decreased from 1.09 on DIM 28 to 0.63 by DIM 321 and the herd variance followed a similar decline from 2.17 to 1.25. However, the PE variance declined from 2.58 on DIM 28 to its lowest value of 1.23 on DIM 197, and then increased to 1.71 on DIM 321. The estimates of heritability for the eight test days ranged from 0.193 to 0.250 with an average of 0.230 (Fig. 5 and Table 4) and a peak on DIM 112. The estimated heritability for average yield across the eight DIM records was 0.361.

**Table 4:** Genetic (below the diagonal) and permanent environment (above the diagonal) correlations and heritabilities (on the diagonal) for selected DIM

Day	28	70	112	154	197	238	280	321
28	0.210	0.992	0.958	0.882	0.749	0.576	0.382	0.204
70	0.998	0.241	0.987	0.935	0.828	0.676	0.497	0.328
112	0.992	0.998	0.250	0.98	0.908	0.787	0.631	0.477
154	0.979	0.99	0.997	0.246	0.973	0.893	0.772	0.641
197	0.959	0.975	0.988	0.997	0.245	0.973	0.898	0.802
238	0.931	0.952	0.971	0.986	0.996	0.240	0.975	0.918
280	0.893	0.919	0.944	0.966	0.984	0.996	0.222	0.983
321	0.846	0.877	0.908	0.936	0.962	0.982	0.995	0.193

*DIM* Days in milk

### Genetic and permanent environment correlations between DIM

The additive genetic correlations between the eight DIM decreased as the interval increased but were always high, ranging from 0.846 to 0.998 (Table 4). The high positive genetic correlation observed across all DIM is useful in these systems, where cows may have milk records only for part of the lactation period, which means that the ability to select for increased full lactation yield does not depend highly on when during the lactation period the data is available for a given cow. PE correlations between DIM followed the same trend as the genetic correlations (Table 4) but with much larger changes as the interval increased, ranging from 0.204 to 0.992.

### Accuracy and bias of GEBV

**Fig. 6:**
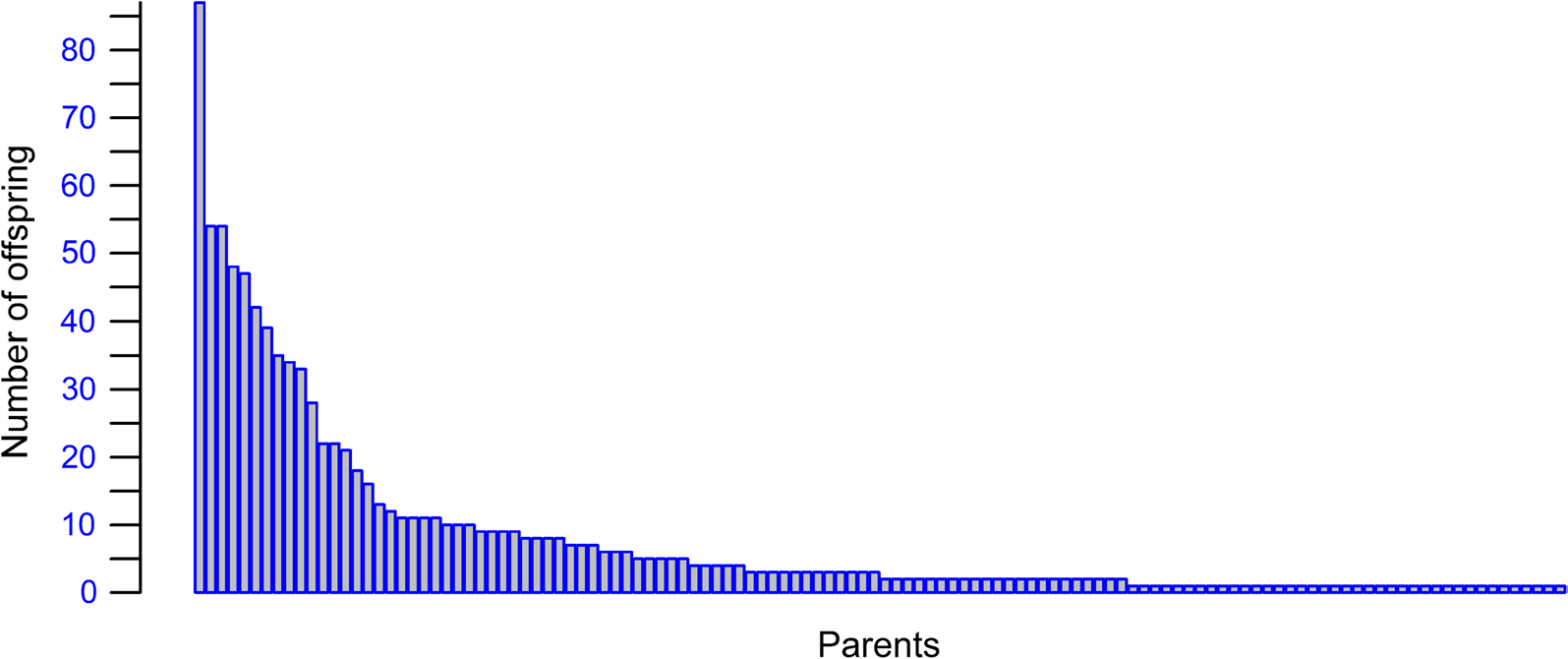
Number of offspring identified to parents. Each bar represents a parent

Of the 3842 genotyped cows and the 661 genotyped bulls, 970 cows (25%) were identified as daughters of 122 bulls (Fig. 6). The number of daughters per bull ranged from 1 to 87, with a median of 3. Because 25% of the genotyped cows were assigned to sires and most bulls had no or very few daughters in the dataset, the validation of GEBV was performed on the genotyped cows, rather than on the bulls.

Validation was undertaken for GEBV ${\widehat{a}}_{\mathrm{\alpha }0}$ obtained from Model 3 (adjusted TD from Model 2-M1) when BC $\times$ ENV was included in the genetic analysis. The results of the validation of GEBV are in Table 5 for method B (i.e. random selection of CDC to form the validation set), which is the method expected to yield the least biased estimates of accuracy. The estimates of the achieved accuracy were 0.196 when validating ${\widehat{a}}_{\mathrm{\alpha }0}$ with the average unadjusted TD, and 0.420 and 0.363, when validating ${\widehat{a}}_{\mathrm{\alpha }0}$ with the average adjusted TD and average TD corrected only for CDC, respectively.

**Table 5:** Achieved accuracies (SE) and regression coefficient (SE) of GEBV for method B

	Target phenotypes		
	Unadjusted TD	Adjusted TD	TD corrected for CDC
Achieved accuracy	0.196 (0.016)	0.420 (0.015)	0.363 (0.015)
Regression coefficient	0.703 (0.123)	1.156 (0.095)	1.013 (0.095)

*SE* Standard error, *TD* Test-day, *CDC* Cattle development centre

The estimate of the achieved accuracy using unadjusted TD is expected to be heavily biased downwards due to the target phenotypes of the validation cows not being adjusted for fixed effects, which inflates the variance of the target phenotypes. The adjusted TD were adjusted for all fixed effects, so that the expected variance of the phenotypes of the validation cows were the same as for the training population. The achieved accuracy (0.420) using adjusted TD was close to the average of the estimates of accuracy of each GEBV derived from their PEV (0.413). Using the TD of validation cows adjusted for CDC resulted in a slightly lower accuracy than the average estimated accuracy based on the PEV. Because the TD adjusted for CDC are not adjusted for other fixed effects, the trait variance is inflated in relation to the variance of the target phenotype, creating a downward bias in the estimate of the achieved accuracy of the GEBV. The slopes of the regressions of the adjusted TD or the TD adjusted for CDC on the ${\widehat{a}}_{\mathrm{\alpha }0}$, were slightly higher than 1.0, indicating that the absolute values of the GEBV slightly underestimated the true breeding values.

GEBV ${\widehat{a}}_{\mathrm{\alpha }0}$ were also obtained from the genetic analysis (Model 3) when BC × ENV was included in the phenotype analysis, and were validated with adjusted TD. The validation accuracy was very similar (0.426) to the validation accuracy obtained when the BC × ENV was included in the genetic analysis (0.420). However, the regression coefficient of TD corrected for CDC on GEBV for ${\widehat{a}}_{\mathrm{\alpha }0}$ was 1.863, which indicated that the absolute GEBV were largely underestimated.

The estimates of the achieved accuracy when method A was used (i.e. random selection of the cows included in the validation sets) (Table 6), were, as expected, substantially higher than those from method B (Table 5). The slope of the regressions for the adjusted TD and the TD adjusted for CDC were also substantially higher than those obtained from method B, whereas the slope of the regression of unadjusted TD was substantially lower.

**Table 6:** Achieved accuracies (SE) and regression coefficient (SE) of GEBV for method A

	Target phenotypes		
	Unadjusted TD	Adjusted TD	TD corrected for CDC
Achieved accuracy	0.143 (0.016)	0.455 (0.014)	0.404 (0.015)
Regression coefficient	0.489 (0.118)	1.214 (0.091)	1.086 (0.092)

*SE* Standard error, *TD* Test-day, *CDC* Cattle development centre

## Discussion

The average TD yield of 7.12 kg/day observed in this study is slightly lower than the official national average of 8.09 kg/day reported for crossbred cows in India [1]. The average TD yield of the high, medium and low production environment classes were 9.64, 6.88, and 4.61 kg/day, respectively, and the estimates of herd effects ranged from − 4.35 to 6.81 kg/day, which illustrate the wide range of production environments across smallholder farms. This shows that it is unwise to think of smallholder farms as being a defined system. Rather, the range of environments in which smallholder farms exist and create for their cows is large. The average yields that we observed here are higher than those reported for similar smallholder crossbred systems in Kenya [5, 8, 15, 16], which suggest better production environments on Indian farms, but also may indicate a difference in the milk production potential of indigenous cattle in India versus Kenya. The average yield was lower than that reported by Pereira et al. [17] for Girolando (Holstein $\times$ Gyr crosses) in Brazil. However, Madalena et al. [18] reported that most of the published research in Brazil does not represent commercial practice, and that average commercial yields of most of the crossbred dairy cattle in Brazil are about the same as those observed for our low production environment class of farms.

A striking feature of our results is the lack of a typical lactation curve for the low and medium production environment classes. A very similar result was found for similar smallholder crossbred cattle in Kenya [8]. For purebred and crosses between *Bos taurus* dairy breeds in Kenya, Wahinya et al. [19] also reported a lack of a typical lactation curve but it occurred at substantially higher production levels. This raises the question: to what extent is the response of the shape of the lactation curve to a lower input environment a function of the genotype of cow?

We present the first results of the genetic evaluation of smallholder crossbred cows in India, in the absence of a structured genetic testing program. The results show genetic variation and heritabilities that can allow genetic improvement of these cows. These results are supported by validation accuracies of GEBV that are almost identical to those predicted by the PEV of the GEBV. These results agree with the results of a similar but substantially smaller study of genetic parameters of smallholder crossbred cattle in Kenya [7]. In both cases, it is a remarkable finding that valuable levels of genetic variation exist in spite of the many challenges due to the data originating from smallholder systems.

Some of the main challenges in smallholder systems include: very small herd sizes (on average, two cows per herd in the current data); unreliable assignment of age and parity when initiating recording on cows of all ages in a system where farmers (mostly) do not keep farm data records, where there is no pedigree recording and inaccurate milk recording with low yields leading to high coefficients of variation; and where the environments experienced by the cows vary enormously as feed supply and quality fluctuate widely in both the short and long terms. These challenges substantially limit the ability of analyses to account for non-genetic effects in ways that are routine in intensive dairy systems. For example, very small herd sizes mean that the usual practice of fitting herd-year-season effects to account for short- and longer-term shared environment effects [20–23] is not feasible. It should be noted that the average herd size reported here overestimates the herd size compared to that normally considered in genetic evaluations because it is based on the number of cows in a given herd that occur in the dataset at some point in time but not necessarily at the same time as other cows under recording. More than 50% of the TD records are for herds with two cows recorded on that day and 20% of the TD records are for herds with a single cow recorded on that day. The data limitations of smallholder systems also prevent the use of more exact genetic models that are often applied in intensive dairy systems. For example, the unreliable assignment of age and parity, coupled with the fact that few cows have data for multiple lactations prevent the application of multi-trait models that treat different lactations as different traits [24–26]. However, as the data accumulate over time, young cows will enter the recording program and will then be followed from first to later lactations, allowing such analyses in the future.

Fitting herd as a random effect rather than a fixed effect, as was done here, improves the accuracy of genetic evaluations when herd sizes are small [21, 27–29]. It becomes critical to fit herd as a random effect when the herd size is very small, because a fixed herd effect would take a very high proportion of the available degrees of freedom and absorb out a high proportion of the phenotypic variation, including the underlying genetic variation. When herd is fitted as a random effect, as long as some cows in the data are related, the relationship matrix separates the additive genetic variance from the herd variance, even if there is only one cow per herd, by providing genetic linkages between herds [30]. As the number of relationships becomes larger, the efficiency of the relationship matrix to separate genetic from herd variation increases. In this regard, an accurate genomic relationship matrix will always contain more and more accurate relationships, and hence be more powerful than the use of a pedigree relationship matrix. With small herd sizes, there will be particular value in having a genomic relationship matrix rather than an initially sparse pedigree relationship matrix. This substantial advantage should persist long after pedigree data begin to accumulate. The immediate value of a genomic relationship matrix, as used here, is that it allows genetic analyses and GEBV to be produced as soon as phenotype data are available rather than waiting for pedigree information to accumulate. The arguments above suggest that genomic relationships may also have longer-term advantages when recording is initiated in smallholder populations versus populations with larger herd sizes, where the large number of cows per herd allows the separation of genetic and herd effects, even with sparse pedigree relationship matrices. This has unexplored implications for the relative value of pedigree recording versus continued genotyping over time in smallholder genetic improvement programs.

In studies such as ours, there is not one compelling piece of evidence that proves a particular model to be superior to the others. The most appropriate model might also depend on how the information is used. In the present case, we presented a subset of a much larger number of phenotypic and genetic models that were explored. Among those presented here, we believe that the most suitable model for the estimation of GEBV is Model 3, based on adjusted TD from Model 2-M1, and including BC $\times$ ENV as a fixed effect in the genetic analysis. In contrast, when advising farmers on the most suitable breed composition for their environment, the BC $\times$ ENV estimates obtained from the phenotype analysis are more appropriate.

As justification for these assertions, Model 2-M1 includes random effects for herd and animal (assumed to be unrelated) in the phenotype analysis, which would be expected to reduce the absorption of animal effects into estimates of the fixed effects. When the adjusted TD were created from the sum of the residuals and random animal effects, excluding the random herd effects, we hypothesised that herd might absorb a proportion of the genetic variance. This was confirmed in the subsequent genetic analysis that yielded a very large drop in additive genetic variance of ${\alpha }_{0}$ from 1.256 to 0.470, compared to the adjusted TD that included the random herd estimates. Then, we examined the relevance of including BC $\times$ ENV effects in the genetic analysis. This was based on the expectation that there are large non-additive genetic effects on the milk production of crosses between *Bos taurus* $\times$ *Bos indicus* cattle in the tropics [2]. Including BC $\times$ ENV in the genetic analysis led to a further increase in the estimated additive genetic variance of ${\alpha }_{0}$ from 1.256 to 1.562, with a concomitant reduction of the PE variance, as expected if including BC $\times$ ENV allows to better account for non-additive crossbreeding effects in the data. The validation analyses of the GEBV from this analysis showed that the GEBV were unbiased and gave validation accuracies almost identical to those derived from the PEV of the GEBV, which gives confidence that this model generates reliable GEBV. The achieved accuracies are much lower than those obtained in intensive dairy industries [31], which reflect the much denser data and denser relationships in intensive dairy systems. Our validation accuracies were similar to those obtained by Brown et al. for a similar smallholder system in Kenya, and those obtained by Powell et al. [32] from a simulation of a smallholder dairy system with an average herd size of 1.58.

As hypothesised and then demonstrated by our results, when breed composition effects are not included in the phenotype models, CDC effects absorb a proportion of the differences in breed composition because of the variation in breed composition between CDC. Thus, the estimates of BC $\times$ ENV effects in the genetic analysis result in underestimates of the differences in breed composition. For advising farmers on the most suitable breed composition, it is better to use the estimates obtained from the phenotype analysis, in which breed composition and CDC effects are fitted simultaneously.

The two-step analysis undertaken here is justified by the large loss of information about non-genetic effects if the data on cows with phenotype but no genotype data are not used. In the absence of any pedigree information, a one-step analysis could, in principle, be applied [33] by assuming that the 4721 ungenotyped cows are unrelated to each other and to the 3842 genotyped cows, and that they all have an average breed composition. The impact on parameter estimates would likely be substantial. However, we were unable to obtain solutions when this was attempted, perhaps because of the very high degree of confounding between herd, PE and additive genetic effects, and because the software that we used could not accommodate the combination of fixed and random effects applied here. The use of a two-step process requires a series of trade-offs in the analyses which means that no set of analyses is ideal. Thus, the goal should be to obtain genotype information on all the cows with phenotypes so that genetic parameters or GEBV and fixed effects can be obtained simultaneously.

The estimates of breed composition effects showed a general trend of increasing yield as the proportion of HF blood increased, and that yields did not increase above intermediate classes (25–50% and 50–75% HF) in the medium and low production environments, which suggests the expression of some heterosis. The estimates for the proportion of Jersey blood had higher SE and the trend of higher yields as the proportion of Jersey blood increased was much weaker than for Holstein. The relatively weak expression of heterosis compared to that expected from the reviews of tropical dairy crossbreeding by Cunningham and Syrstad [2] may reflect that the literature is based on defined crosses such as F1, F2 and first backcross, whereas smallholder crossbreds predominantly result from many generations of crossing. After analysing published data, Rutledge [34] concluded that *Bos taurus* $\times$ *Bos indicus* dairy crosses exhibited a much larger loss of heterosis in second and later generation crosses than expected from the loss of heterozygosity caused by recombination. He postulated that this was due to the breakup of epistatic interactions that had evolved within *Bos taurus* and within *Bos indicus.* We are currently undertaking a range of alternative analyses of breed composition effects, including analyses that explicitly fit heterosis, to better understand the effects of breed composition and their interaction with production environments in Indian smallholder systems.

Our results are a first attempt to obtain useful GEBV and breed composition effects for these complex and diverse smallholder systems. While the estimates that we obtained should be sufficiently reliable to initiate genetic improvement, many issues require further exploration to improve these estimates as the data accumulate. Apart from the obvious need to extend these results to other traits that affect the profitability and sustainability of these systems, we expect that substantial genotype-by-environment interactions (G $\times$ E) may be present. We acknowledged this when fitting breed composition effects for three production levels but there is no allowance for G $\times$ E that affect additive genetic effects contributing to the estimation of GEBV. In addition, while production level as defined here effectively integrates multiple environmental influences on production, there are other environmental factors, such as heat stress, that vary over time and location that could significantly affect the genetic merit of different animals for production in different environments [35]. A full optimisation of genetic analyses and genetic improvement in these systems will require many years of substantial research investment, as has already been the case for the intensive dairy systems worldwide.

## Conclusions

Our results show that, in spite of the many challenges of smallholder systems and data, there is substantial additive genetic variation for milk production in smallholder crossbred dairy systems in India. A combination of smallholder data recording and SNP genotyping can be used to generate GEBV, allowing immediate genetic improvement of bulls entering AI. Using the same data, the performance of the cows of different breed compositions can be determined in different production environments, which makes it possible to provide better advice to smallholder farmers on optimum breed composition for their environment. In the same way that genetic improvement in intensive dairy systems has benefited greatly from the large volume of research since progeny testing first allowed effective genetic improvement to be achieved, a much deeper understanding of smallholder systems, along with the genetics and value of traits in such systems, will enable more effective genetic improvement in smallholder systems to evolve in the future.

## Data Availability

The data generated specifically for this study are available from M. Swaminathan mswami@baif.org.in upon reasonable request. Reference data are available from public and private databases as described in the paper.
